# Glycemic index values of traditional Kenyan foods: the missing link in the effectiveness of dietary approach in the prevention and management of diabetes mellitus in Kenya

**DOI:** 10.4314/ahs.v21i2.29

**Published:** 2021-06

**Authors:** Rebecca Ebere, Jasper Imungi, Violet Kimani

**Affiliations:** 1 Meru University of Science and Technology, Department of Food Science; 2 University of Nairobi, Department of Food Nutrition and Technology; 3 University of Nairobi, School of Public Health

**Keywords:** Glycemic index, glycemic load and glycemic response, Kenya

## Abstract

**Background:**

Glycemic index (GI) measures postprandial blood sugar after consumption of carbohydrate-rich foodstuff. Kenya is yet to fully embrace this concept in prevention and management of diabetes mellitus.

**Objective:**

To review and tabulate GIs of locally consumed foods in order to improve dietary management of diabetes mellitus.

**Methodology:**

A literature search was conducted using Google scholar and PubMed databases which identified 7 articles on glycemic index values of Kenyan foods published between 2002 and 2020. Two articles failed to meet the inclusion criteria and five proceeded for review. Key search words used included GI, glycemic load and glycemic response combined with Kenya. The data was reported depending on whether the testing involved healthy individuals or patients suffering from diabetes mellitus.

**Results:**

Nine individual foods and 7 mixed meals were identified. Low GI foods included beans and whole maize ugali consumed alongside cowpea leaves. High GI foods included whole maize *ugali* eaten with beef, boiled rice, boiled cassava and cassava-sorghum *ugali* eaten with silver fish.

**Conclusion:**

Proper meal mixing is important in diabetes management. Cowpea leaves and beans possess GI lowering potential. This information can be used to improve guidance on food choices for diabetes patients.

## Introduction

This paper is addressing itself to the nutritional challenge faced by the dietary approach in the prevention and management of diabetes mellitus in Kenya. Diabetes mellitus is a chronic medical condition in which a person's sugar level rises above normal. If left uncontrolled, the excess sugar may damage the nerves and blood vessels resulting in amputation, blindness, renal failure, infertility in men, heart disease and stroke 1.

Diabetes mellitus is prevalent worldwide. In the year 2019 the global prevalence of diabetes was estimated to be 9.3% (463 million people), this was projected to rise to 10.2% (578 million) and 10.9% (700 million) by the years 2030 and 2045 respectively 2. In the year 2015 an estimated 1.6 million people died from diabetes in Kenya 3. In 2016, non-communicable diseases including diabetes mellitus accounted for about 27% of all deaths in the country 4.

There are many risk factors for diabetes mellitus including genetics 5, lack of physical exercise, excessive consumption of alcohol and smoking of cigarettes 6. Diabetes management aims at keeping glucose levels in the blood closer to the normal level as much as possible. Dietary approach has been clinically adopted in the prevention and management of diabetes to ensure healthy blood sugar level 7. In addition to dietary approach, exercise and use of treatment drugs are also widely applied^8^. However many diabetic patients cannot afford the treatment due to the high cost resulting in complications and high mortality 9. In addition, management of diabetes also involves limiting alcohol consumption; avoiding cigarette smoking; regular monitoring for associated complications involving eyes and feet, blood sugar, blood pressure as well as risks for kidney and cardiovascular diseases 10. Kenya government is facing a number of challenges in prevention and management of diabetes. These include limited resources, a shortage of adequately trained healthcare personnel and lack of necessary equipment subsequently leading to lack of awareness and routine screening 11.

Dietary approach to the prevention and management of type 2 diabetes is a common practice in Kenya although patients' adherence to recommendations still poses a challenge 12. Current dietary prescription is mainly based on the amount of food served to diabetic patients, a macro-nutritional approach commonly known as the healthy diabetes plate, the plate method or simply “diabetic plate”. It guides how much starchy food should be served in relation to non-starchy vegetables and proteins without considering how the meal affects postprandial blood sugar 13. A key shortcoming with such an approach is the fact that carbohydrate-rich foods differ in postprandial blood sugar response. This means that a similar amount of different foodstuff cause different responses in terms of postprandial blood sugar. For example, 50g available carbohydrate from cassava is different from 50 g available carbohydrate from sweet potato in their potential to raise blood sugar levels 14.

Glycemic index (GI) has widely been used to measure blood sugar responses after consumption of a carbohydrate-rich food in order to take into account such differences. GI is computed by dividing the area under the blood glucose response curve above the fasting blood sugar level after consuming 50 g available carbohydrates from a test food by area under the blood glucose response curve above the fasting level after consuming 50 g available carbohydrates from a standard or reference food and multiplying the resulting value by 100. White bread or glucose are used as reference/standard foods and they are normally assigned a GI of 100 15. Thus foods have been categorized into low (<55), medium (55–70) and high (>70) GI with glucose as a standard or values of <60, 60–85 and >85 representing low, medium and high GI respectively with white bread as a standard/reference food 16. High GI foods result in a greater blood glucose response and long-term consumption may lead to type 2 diabetes 17 as opposed to low GI foods 7. However, consuming a high GI food alongside a low GI food or other accompaniment has been shown to reduce the overall blood glucose response of a high GI food. For example the GI of rice has been reduced by beans 18, 19.

It is important to note that even low GI foods can result in higher blood glucose response and high GI foods may cause low response after consumption depending on the quantity of food consumed. This is better explained by glycemic load (GL) which accounts for both the quality as measured using GI and the amount of food consumed. GL is computed as follows; GL = GI/100 x available carbohydrates (total dietary carbohydrates - dietary fiber). Using this approach, foods can be classified into low (1–10), medium (11–19) and high (≥20) GL 16 and this concept allows the right amount of food to be served for effective management of blood sugar.

Without taking into consideration the GI of the foods, there is a possibility of putting a diabetic person at risk of spiking their blood sugar using the macronutrient approach to management of diabetes. The GI of foods can however be manipulated in various ways to suit the needs of people suffering from diabetes mellitus. For example the GI has been found to vary depending on the nature of carbohydrates 20, dietary fiber, 21, 22, 23, other macronutrients present in the diet 21, 24, 25, presence of micronutrients 24, preparation, processing and storage 20,26, 27, botanical origin and variety 17, 18, 28, 29, 30, 31, presence of phytochemical 28, 32 as well as an accompaniment to the staple or mixing different foods into meals 18, 19.

Despite the advantage posed by GI, adequate information on GI values of most traditional foods consumed by Kenyans is missing. This creates a challenge for the practitioners with regard to the selection of the appropriate diet for diabetics. The available international table of glycemic indices of foods 7, 33 currently being adopted in selecting the diet for diabetics is limited in terms of the commonly consumed Kenyan foodstuff. As a way of preventing and managing diabetes through a dietary approach, it is important to identify and promote consumption of culturally acceptable, locally available and affordable staple foods 34. This paper therefore, reviewed glycemic indices of various traditional carbohydrate-rich foods consumed in Kenya with an aim of improving the knowledge base and the possibility of increasing their utilization in the prevention and management of diabetes mellitus.

## Methodology

An electronic database literature search was conducted on Pubmed and Google Scholar on studies published between 2002 when the first international Table of glycemic index values was published 7 to April 2020. Inclusion criteria were studies published in English, involving human studies and following standardized methods. Exclusion criteria were studies not published in English, in vitro studies, those involving animals and those that did not follow standard methods 33. The search terms included glycemic index, glycemic load and glycemic response combined with Kenya. Glycemic index values listed in previous tables 7 were also included.

Final data were divided into two tables comprising of values derived from healthy subjects and values derived using individuals suffering from type 2 diabetes mellitus. To ensure uniformity in presentation of results, those presenting values in mean ± SD had the standard deviation value divided by square root of sample size to present all the results as mean ± SE. Values that were tested using white bread as a reference and then converted to glucose scale do not indicate the SE. Values that were tested used white bread as a reference and then converted to glucose scale do not indicate the SE. Results of available carbohydrate are presented on dry weight basis unless otherwise indicated against the foodstuff. Also, since both glucose and white bread are used as reference foods, a conversion factor of 70/100 (0.7) was used to convert from white bread scale to glucose scale. Hence the glucose scale has been used for final reporting as recommended to avoid possible confusion in the interpretation of the results 7, 33. Carbohydrate content was obtained from the reference papers. GL values were calculated as the product of the amount of available carbohydrate in a specified serving size and the GI value (using glucose as the reference food), divided by 100 7, 33.

## Results

Glycemic index values cereal-based individual foods/meals tested on healthy individuals follow the order: boiled white rice > whole maize ugali with beef >whole maize ugali with silver fish= rice with beef> whole maize ugali (plain)=rice with beans>whole maize ugali; cowpea leaves. Bean stew had a GI of 44 while roots and tubers' products followed the following order: cassava -sorghum ugali with silver fish>boiled cassava> Cassava-sorghum ugali with cowpea leaves> sweet potato. Low GI was observed in beans and whole maize ugali consumed alongside cowpea leaves. These results are shown in Table 1.

**Table 1:** Glycemic indices of some Kenyan foods tested on healthy individuals (n≥7)

S/N	Food type	Available carbohydrate (% dwb)	Serving size (g) containing 50g carbohydrate (wwb)	GI (mean ± SE) Ref: glucose
**Cereal-based products**				

1	Whole maize *ugali;* plain	92.09±0.27	160	62±8.90
2	Whole maize *ugali;* silver fish	92.09±0.27	148; 30	69.1±10.00
3	Whole maize *ugali*, cowpea leaves	92.09±0.27 18.86±0.05	158; 50	45±6.60
4	Whole maize *ugali;* beef	92.09±0.27 Assumed 0	160; 150	71±6.70
5	Boiled white rice (*mwea pishori*)	25.98±0.12 wwb	192	77±5.69
6	Rice (*mwea pishori*); beef	25.98±0.12 wwb Assumed 0	192; 150	69±7.70
7	Rice; beans	25.98±0.12 wwb	142; 80	62±5.16
	Mwea pishori rice; rose coco beans	16.03±0.15 wwb		

**Legumes**				

**1**	Bean stew	16.03±0.15 wwb	312	44±10.00

**Roots and tubers' products**				

1	Sweet potato	90.04±0.03	168	64.54±7.11
2	Boiled cassava	78.81±0.45	175	74.10±6.31
3	Cassava-sorghum *ugali;* cowpea leaves	91.79±0.12 18.86±0.05	164; 50	69±9.01
4	Cassava-sorghum *ugali;* silver fish	91.79±0.12 34.45±0.0	157; 30	83±12.01

Ref: 14, 18, 35

For the specific foods tested on individuals suffering from type 2 diabetes mellitus, the order of GI values was as follows: boiled white rice>maize meal porridge>millet flour porridge>boiled and salted cassava. These results are shown in Table 2.

**Table 2:** Glycemic indices of some Kenyan foods tested on individuals who were suffering from type 2 diabetes mellitus (n≥7)

S/N	Food type	Available carbohydrate	Serving size (g) containing 50g carbohydrate	GI (mean ± SE) Ref: glucose
**Cereal-based products**				

1	Boiled white rice (unspecified variety)	42g available carb/150g serving	150	112
2	Maize meal porridge/ gruel	38/50g	50 g (dry)	109
3	Millet flour porridge/ gruel	unspecified	unspecified	107

**Roots**				

4	Cassava, boiled, with salt	27g/100g	100	46

Ref: 7

## Discussion

Kenyan population consumes a variety of foods including snacks, thin porridges (uji), dishes from rice, maize, legumes, meat, fish, eggs, poultry, blood dishes, vegetable, roots and bananas, stiff porridge (ugali), flat bread (chappati), mashed dishes, deserts and sauces. Staple food is mainly maize and other cereal grains such as rice, wheat, millet and sorghum depending on the region. These staples are usually consumed alongside a variety of meats, legumes and/or vegetables 36.

### Glycemic index of cereal-based food products/meals

The predominant cereals consumed by the Kenyan population include maize (Zea mays), rice (Oryza sativa) and wheat (Triticum aestivum) 37. Maize is consumed in the form of a thin (uji) or stiff/thick (ugali) porridge. The stiff porridge is usually consumed alongside an accompaniment 18, 35, 36. The same applies to many rice and wheat products 36. Whole milled maize, sorghum (Sorgum bicolor) and finger millet (Eleusine coracana) have been recommended in some parts of the country for preparing ugali (stiff porridge) and uji (thin porridge) for people suffering from non-communicable diseases including type 2 diabetes mellitus but also plain cassava alone or mixed with finger millet, sorghum or both can be used to prepare ugali 38.

A moderate GI of 62 has been reported in a study conducted on healthy individuals in Kenya (Table 1). A GI of 94 was reported for stiff porridge from Malawi which has a moisture content of 76.54% as opposed to 66% in the Kenyan study 39, 40, 41 which may have influenced the extent of starch gelatinization. Processing methods have been found to influence the GI 42, 43. For instance, cooking in excess water resulted in a higher rate of starch digestion while cooking in limited water may allow starch granules to rearrange and interact together limiting the rate of digestion 43. Whole maize ugali consumed in Tanzania had a GI of 71 44. The differences in GI could also be as a result of possible differences in maize varieties 32 which were not documented in these studies. The possible differences in varieties would also mean differences in amylose to amylopection ratio which influence formation of resistant starch and consequently starch digestibility 20. In addition, similar foods made from flour milled by different methods have been found to vary in terms of GI 27. The particle sizes and milling methods were not documented in these studies.

As opposed to silver fish (Rastrineobola argenteus) and beef, cowpea leaves (Vigna unguiculata) lowered the glycemic index of whole maize meal ugali. A low GI of 45 was reported. Cowpea leaves contain significant amounts of protein, fiber, prebiotics, fat, iron, calcium, phosphorus, magnesium, iodine, potassium and sodium^45^, ^46^. In addition, cowpea leaves contain phytochemicals including polyphenols, tannins, saponins and flavonoids^46^. Most of these components have been associated with lower GI, for example magnesium 47, polyphenols 32, and tannins 48. In addition, dietary fiber and magnesium intakes may have reduced insulin resistance^49^. Generally a low GI diet has been associated with high intakes of micronutrients, fat and sodium 24 as well as other macromolecules present in the diet 22, 29.

Apart from cowpea leaves, other vegetable dishes that can be consumed alongside ugali and whose impacts on GI are yet to be determined include stir fried kales; cabbage; jute mallow and pumpkin leaves; pumpkin leaves; spider plant, amaranth and African nightshade leaves; vine spinach; stir-fried amaranth leaves; mashed pumpkin and African nightshade leaves; stinging nettle leaves; stewed cowpeas leaves and jute mallow leaves; stir-fried spinach; sweet potato leaves; stewed mushrooms in peanut butter; potato curry; peas and brinjal curry 36. On the other hand consumption of ugali accompanied by beef or silverfish popularly known as omena in Kenya, resulted in an increased glycemic index of 69 (Table 1). Although both were rich in protein which reduce postprandial glucose response 25 and fat that also lowers the GI 24 the GI was increased. This could be explained by the fact that the amount of ugali in relation to accompaniment is usually large. The large proportion of carbohydrates could have outweighed the GI lowering effect of protein or fat 22. In addition, beef has been associated with decreased insulin sensitivity 50.

The impact of the following meat, fish and egg dishes on GI of ugali has not yet been documented: stir fried goat meat; stir fried beef; Swahili spiced beef stew; minced meat balls; stewed dried fish; fried tilapia; Hydrabadi biryani; stewed Nile perch; stewed goat meat; camel meat; sheep tail fat; beef, maize and wheat flour mix; okra meat dish. In addition the effect on GI of sautéed termites, fried egg, omelette, Spanish omelette and poultry including stewed chicken; fried chicken; stewed quails and stewed guinea fowl as well as blood dishes including cow blood, beef and cow fat; cow blood with sour milk; blood cooked in fresh milk; fresh blood and fresh milk 36.

Aside from ugali prepared from whole maize flour, ugali can also be prepared from maize and finger millet flour; maize, red sorghum and finger millet flour; refined maize flour; red sorghum, maize and finger millet flour; cassava flour; cassava, finger millet and sorghum flour; finger millet flour as well as banana and maize flour. Ugali can also be prepared from any of these flours and cooked using sour milk instead of water 36. The GI of these preparations is yet to be determined. The GI of gruel/thin porridge (uji) prepared from maize meal porridge and millet meal was 109 and 107 respectively (Table 2). However it is important to note that these were results conducted on individuals suffering from type 2 diabetes mellitus. A recent study conducted on glycemic responses from gruel prepared from finger millet and white maize flours did not find significant difference between the two samples in terms of postprandial glycemic response although this study did not calculate the GI of these samples 51. Other cereal-based products with unknown GI and are usually consumed alongside same accompaniments as ugali and rice include wheat products such as white chapati, brown chapati, roti (Indian chapati) and bhature (fried Indian bread). The GI is also yet to be determined for the following maize dishes: ashir; fresh beans and maize; dried maize and beans; sautéed maize and beans; dehulled maize and beans; crushed maize 36.

Plain boiled white rice had a GI of 77 which was lowered to 69 and 62 when consumed with beef and beans respectively (Table 1). The unspecified white rice variety had a GI of 112 tested on type 2 diabetes patients (Table 2). The difference could be attributed to the different rice varieties 29, 31 and possibly between starches obtained from different locations 52. In addition, the unspecified white rice variety in Table 2 could also be from the fact that the test participants were diabetes patients^53^. Both beef and beans lowered the GI probably due to their protein content 21, 25. The protein source could also be playing a significant effect with better effect observed from proteins of plant origin 54. In addition, the GI lowering effect from beans could be resulting from presence of fiber 21, 55, 56, polyphenols 57 or resistant starch and the beans cell walls which modulate starch gelatinization thereby reducing enzymatic hydrolysis 58. In addition beans being a lente-carbohydrate possess a low GI which could have diluted high GI of rice.

Rice dishes with unknown GI include spiced rice (pilau), Swahili biryani rice, onion fried rice, potatoes in rice, steamed rice and rice with milk. Rice in Kenya can be consumed alongside same accompaniments discussed above for ugali. Other cereal-based products with unknown GI and are usually consumed alongside same accompaniments as ugali and rice include wheat products such as white chapati, brown chapati, roti (Indian chapati) and bhature (fried Indian bread). The GI is also yet to be determined for the following maize dishes: ashir; fresh beans and maize; dried maize and beans; sautéed maize and beans; dehulled maize and beans; crushed maize 36.

### Glycemic index of legumes

The GI of beans was 44 (Table 1). The factors that could have contributed to the low GI of beans have been discussed above alongside rice and beans meal. Other legume dishes whose GI data is still missing include lentil stew; green gram stew; bean stew with milk and cream; black bean stew; pigeon peas stew; skinned bean stew; stewed split dal; chick peas curry; sautéed red kidney beans and red sorghum; red sorghum, beans and teff flour 36. These stews can be consumed alongside ugali, rice or chapatti and their effect on GI of these staples is yet to be documented in Kenya.

### Glycemic index of roots and tubers products

In a study conducted on healthy individuals, the GI of sweet potato (Ipomoea balatas L.) was 65 and that for cassava (Manihot esculenta Crantz) was 74 although the two values were not found to be statistically different (Table 1). Jamaican boiled sweet potato had a GI of range of 41± 5 to 50±3 (SEM) in ten different varieties^59^. The differences could be attributed to the origin or geographical location 52, 60. Bangladesh sweet potato recorded a GI of 191±66 (SD) in a study conducted on individuals suffering from type 2 diabetes mellitus 53. A GI of 74 reported in the Kenyan study with regard to cassava was high. This is in agreement with a Caribbean study conducted on healthy individuals that reported a GI of 94±11 (SEM) 26. Cassava boiled in salt and tested on individuals suffering from type 2 diabetes reported a GI of 46 although the portion size administered may not be equivalent to 50 g available carbohydrate. In addition a higher sodium intake has been associated with a low GI 24 although higher salt intake may not be encouraged due to associated detrimental health effects. Cassava has also been used in preparation of ugali. This is usually from flour prepared using fermented and sundried cassava pieces mixed with either finger millet, sorghum or both 36. Ugali prepared from cassava and sorghum consumed alongside cow pea leaves had a GI of 69 (moderate) while when consumed with silver fish the GI was 83.The effect of cowpea leaves and silver fish on GI have been discussed earlier with regard to whole maize meal ugali. In both scenarios cowpea leaves lower the GI as opposed to silver fish.

However differences can be noted between whole maize meal *ugali* and cassava-sorghum ugali with whole maize meal *ugali* having a lower GI when consumed with either cowpea leaves or silver fish. A study on sorghum chips reported a high GI (>70) 54 and this together with a high GI for cassava in the Kenyan study could partially explain the higher GI of *ugali* prepared from cassava-sorghum as opposed to that from whole maize meal. A separate study found most sorghum-based food products to have lower GI than their respective wheat or rice-based foods 30. It is important in future studies to note the sorghum variety and the ratios used in composite flour. For example the ratio of sorghum to cassava flour used in preparation of ugali is usually very low 18, 38.

Other foods in this category whose GI is yet to be determined locally include arrowroots; yam stew; sweet potatoes with peanut butter; arrowroot stew; pan fried arrowroots; pan fried sweet potatoes; potato bhajia and potato chips as well as mashed dishes including fresh maize, potato and pumpkin leaves mash; maize, beans, potatoes and pumpkin leaves mash; black beans, green bananas and potatoes mash; green maize and sweet potatoes mash; sweet potatoes and de-hulled black beans mash; mashed cassava and pigeon peas; mashed potatoes; enriched mashed potatoes; mashed beans and potatoes; mashed sweet potato and black beans 36.

### Other foods with unknown glycemic index

In addition to the foods listed and discussed above, the GI of following foods is still missing include: green bananas and stewed green bananas with meat; mashed bananas plain; enriched mashed bananas; enriched matoke; pumpkins with peanut butter; fresh maize; potato and pumpkin leaves; mashed pigeon peas and green maize and common snacks (fried dumplings; Swahili doughnuts; enriched East African doughnuts; East African doughnuts; meat samosa; vegetable samosa; pancakes; drop scones; Qita; mkate kuta; egg toast; oatmeal; pumpkin and coconut milk). These common snacks are mostly consumed alongside mixed tea. Desserts and sauces (sweetened pumpkin and coconut milk; semolina and nuts; diluted yoghurt; groundnut sauce) 36 could also impart on GI and their effect is not documented.

### Use of GI data in computing GL and appropriate food portion size in diabetes management

As described earlier, foods can be classified into low (1–10), medium (11–19) and high (≥20) GL.

Consider an example of GI of beans (rose coco) which is estimated at 44

Using the formula: GL = GI/100 x Available carbohydrates.

Assuming the aim is to give a patient a glycemic load of 10 (low GL), what portion size should the patient be served?

GL = (44/100) x Available carbohydrates

10=0.44 available carbohydrates

Available carbohydrates equivalent to a GL of 10 = 22.72g

Available carbohydrate in beans was 16% (wwb)

Therefore to attain a GL of 10, the serving portion size should be (22.72 x 100) divided by 16 = 142 g of beans.

## Conclusion

Data on glycemic index values for Kenyan local foods is limited. This paper calls for the need to build a data base of the GI of traditional Kenyan foods. Various factors influencing GI especially of common staples should be exploited in dietary prevention and management of diabetes. These may include appropriate mixing of foods into meals taking into consideration the food types and ratios, processing/cooking methods as well as food variety.

### Key recommendations in the application of dietary approach in prevention and management of diabetes in Kenya

1. GI of local foods should be determined and adopted as a key measure of nutritional quality of foods
2. The public should be educated on the how to combine various foodstuffs into meals depending on their GI
3. Various factors affecting the GI could be explored with a view of lowering the GI of common staple foods
4. Policy makers should consider the inclusion of GI information on the existing book; “Kenyan Food Recipes, a Kenyan recipe book of common mixed dishes with nutrients values”.
